# Strong evidence that callous–unemotional traits are not related to risk-taking task performance

**DOI:** 10.12688/f1000research.14623.1

**Published:** 2018-04-26

**Authors:** Luna C. M. Centifanti, James Negen

**Affiliations:** 1 University of Liverpool, Liverpool, UK; 2 University of Durham, Durham, UK

**Keywords:** Callous–unemotional traits, risk taking, decision making, driving, gambling, Bayesian, re-analysis

## Abstract

A hypothesized association between callous–unemotional (CU) traits and risk-taking may account for the link between CU traits and real-world risky behaviors, such as illegal behavior. Prior findings show that reward and punishment responsivity differs in relation to CU traits, but is not associated with general risk-taking. However this has only been examined previously with one task, only with a frequentist framework, and with limited interpretation. Here, we expand to another task and to Bayesian analyses. A total of 657 participants (52% female) completed the Inventory of Callous–Unemotional Traits, the Balloon Analogue Risk Task (essentially a gambling task), and the Stoplight driving task, which repeatedly presents participants with riskier or less risky choices to make while driving. We found strong evidence for the null model, in which there is no relation between the two risk-taking tasks and CU traits (R ^2^ = 0.001; BF _10_ = 1/60.22). These results suggest that general risk-taking does not underlie the real-world risky behavior of people with CU traits. Alternative explanations include a different method of valuing certain outcomes.

## Introduction

Callous–unemotional (CU) traits are an aspect of psychopathy, which includes traits such as callously using others for one’s personal gain, a lack of caring for society’s values and lacking emotional depth ^1^. Risk-taking includes choosing behaviors with uncertain outcomes (but possibly higher rewards) over behavior with more certainty in its rewards ^2^. Here, we show that the two are unrelated when measured in a laboratory setting.

This is surprising for three reasons. First, a variety of risky real-world behaviors and illegal behaviors ^1^—themselves risky—are associated with CU traits (e.g., substance use, sexual risk-taking) ^3– 7^. Second, there is a difference in reward and punishment responsivity in relation to CU traits ^4, 6, 8– 12^. For example, in a test of gambling, the Balloon Analogue Risk Task (BART), CU was related to weaker reward responsivity, in that adolescents with these traits failed to show an increase in risk-taking following successful (rewarded) trials ^4, 13^. Third, CU traits are one aspect of a cluster of traits known as psychopathy, which is associated with risk-taking ^14– 16^.

These data were originally collected as part of a study about the influence of peer presence on risk-taking behavior, with two laboratory tasks conducted ^13, 17^. CU traits were measured as a potential moderator. Results on the relationship between CU traits and a gambling task have been previously reported using frequentist methods, but the null finding failed to be interpreted ^13^. Here, we re-analyze the data in a Bayesian framework, allowing for the relationship between CU traits and gambling to be interpreted. In addition, for the first time, we report our findings on the association between CU traits and a driving risk-taking task ^17^.

## Methods

### Participants and tasks

A total of 675 people (52% female; 16–18 years of age) from six schools in Northwest England participated in 2010. Heads of schools acted *in loco parentis*, and verbal consent was obtained to ensure privacy, which was approved by the ethics committee, within the schools where the research was conducted. Ethical approval was given by the University of Central Lancashire to the first author (PSY0809122). Complete information about the sample and recruitment can be found in a previous report ^17^.

A total of 657 participants produced usable data on all three measures reported here. The Inventory of Callous Unemotional Traits (ICU) ^18– 20^, a self-reporting questionnaire, was used to assess CU traits. The BART ^21^, where participants can repeatedly gamble by pumping a balloon for greater reward but risk popping it and receiving no reward, was one measure of risk-taking. The Stoplight driving task ^22^, where participants repeatedly choose to either enter yellow/red lights and risk time-consuming crashes or stop and then proceed on the green light, was also given in counterbalanced order as an additional risk-taking task. All three are standard choices that have been validated ^19, 21, 23^. At the time of writing, the ICU and BART tasks can be obtained online, and the Stoplight can be obtained by contacting the authors ^22^. Unrelated to the aims of the present study, participants were asked to bring two friends of the same gender and completed the tasks either in their presence or not.

### Statistical analysis

MultiLevel Data Manipulations were conducted in MLwiN 2.30 (University of Bristol, 2014), resulting in an outcome variable for each task that was adjusted to be equated across peer group membership. Descriptive statistics, zero-order Perason correlations and p-values were calculated using JASP 0.8.2.0 ^24^. An online tool was used to calculate Bayes factors ^25^.

## Results

**Figure 1.**
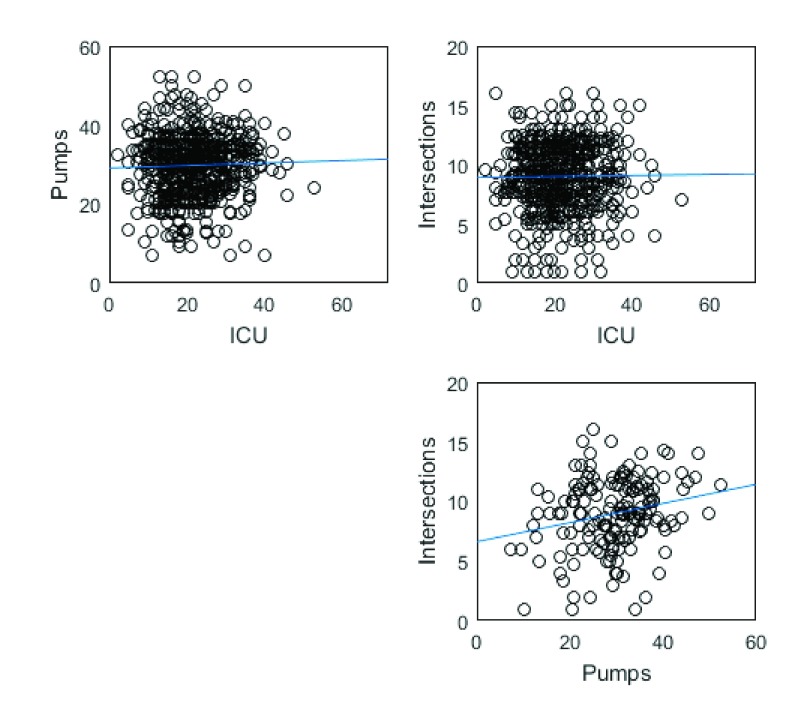
Scatterplots of the relations among the three variables. Scores were adjusted for peer-level clustering, since participants were recruited with two friends.

Figure 1 shows scatterplots of the relations among the three variables. There was a significant zero-order correlation between the tasks, *r*=0.22, *p*<0.001, but not between the ICU scores and either the BART, *r*=0.033, *p*=0.397, N=657, BF _10_=1/8.09 or the driving task, *r*=0.013, *p*=0.738, N=672, BF _10_=1/11.00. More importantly, a multiple linear regression, with the risk-taking tasks predicting ICU scores, showed no significant relation to the BART, β=0.033, *t*=0.824, *p*=0.410, or the driving task, β= -0.000, *t*= -0.012, *p*=0.990. The overall fit was *F* (2, 654)=0.359, *p*=0.698; *R ^2^*=0.001, *R*=0.033, N=657, BF _10_=1/60.22. In a Bayesian analysis, this is considered strong evidence for the null hypothesis ^25, 26^.

We also examined the comparability of our sample to others. The mean ± SD for total ICU (21.62 ± 7.85) was comparable to previous community and at-risk samples. For example, our scores were similar to those from a community sample (male, 25.25 ± 7.90; female, 21.76 ± 9.4) ^27^, as well as to youths from a residential facility (25.74 ± 7.95) ^28^.

Dataset 1. Subject demographic information, together with Inventory of Callous–Unemotional Traits score and results of the tasks http://dx.doi.org/10.5256/f1000research.14623.d201818
Data are provided in raw form and peer-level adjusted format within the same spreadsheet ^29^. Condition: peer present, 1; peer absent, 0; Subject ID, anonymized participant ID number; Female: female gender, 1; male gender, 0; Age, age in years; BART Pumps AdjAvg_raw data, adjusted average pumps; Peer group level adjusted BART Pumps, peer-group level-adjusted adjusted average pumps; Peer ID, peer group membership ID number; Stoplight Intersections_raw data, number of intersections entered on the Stoplight driving task; Peer group level adjusted Stoplight, peer-group level-adjusted number of intersections entered on the Stoplight driving task; Total ICU, number of CU traits using the Inventory of Callous-Unemotional Traits. Click here for additional data file. Copyright: © 2018 Centifanti LCM and Negen J 2018 Data associated with the article are available under the terms of the Creative Commons Zero "No rights reserved" data waiver (CC0 1.0 Public domain dedication).

## Discussion

The results of this study rule out a specific theory about why CU traits are related to risky real-world behaviors including illegal behavior. People with CU traits are not more likely to engage in risky behavior in a lab setting, so real-world risky behaviors are unlikely to be driven by risk-seeking for its own sake. More broadly, this is a worked demonstration that differences in reward and punishment responsivity on a task do not necessarily imply differences in overall risk-taking, even in the same dataset. On the basis of previously reported findings ^13, 17^ and our re-analyses, we conclude that these two concepts should not be used interchangeably in interpreting risk-taking results.

There are potential alternative explanations for why people with high CU traits tend to do risky things, like having unprotected sex. For one, they may simply place different values on the outcomes of catching a disease and/or seeking bodily sensations. However, an interaction between CU traits and antisocial behavior (i.e., conduct disorder) has shown effects on laboratory risk-taking ^23^. One broad possibility is that CU traits do not operate singly, since psychopathy is multifaceted, and some factors of psychopathy appear to be more reliably related to risk taking than others ^14, 30^.

People who engage in antisocial behavior suffer legal, educational and socio-economic consequences ^31^, and we know CU traits predict antisocial behavior ^32, 33^. Thus, further research is needed to understand the mechanisms by which people with CU traits (i) engage in antisocial behavior, and (ii) fail to care about the consequences of their behavior on themselves and on other people. The present study sheds light on one part of this, by showing that one obvious idea of how CU traits and illegal behavior relate is not tenable.

## Data availability

The data referenced by this article are under copyright with the following copyright statement: Copyright: © 2018 Centifanti LCM and Negen J

Data associated with the article are available under the terms of the Creative Commons Zero "No rights reserved" data waiver (CC0 1.0 Public domain dedication). 

**Dataset 1. Subject demographic information, together with Inventory of Callous–Unemotional Traits score and results of the tasks.** Data are provided in raw form and peer-level adjusted format within the same spreadsheet ^29^. Condition: peer present, 1; peer absent, 0; Subject ID, anonymized participant ID number; Female: female gender, 1; male gender, 0; Age, age in years; BART Pumps AdjAvg_raw data, adjusted average pumps; Peer group level adjusted BART Pumps, peer-group level-adjusted adjusted average pumps; Peer ID, peer group membership ID number; Stoplight Intersections_raw data, number of intersections entered on the Stoplight driving task; Peer group level adjusted Stoplight, peer-group level-adjusted number of intersections entered on the Stoplight driving task; Total ICU, number of CU traits using the Inventory of Callous-Unemotional Traits.

DOI: 10.5256/f1000research.14623.d201818 ^29^

## References

[1] Frick PJ Ray JV Thornton LC: Annual research review: A developmental psychopathology approach to understanding callous-unemotional traits in children and adolescents with serious conduct problems. 2014;55(6):532–548. 10.1111/jcpp.12152 24117854

[2] Mills B Reyna VF Estrada S: Explaining contradictory relations between risk perception and risk taking. 2008;19(5):429–433. 10.1111/j.1467-9280.2008.02104.x 18466401

[3] Baskin-Sommers AR Waller R Fish AM: Callous-Unemotional Traits Trajectories Interact with Earlier Conduct Problems and Executive Control to Predict Violence and Substance Use Among High Risk Male Adolescents. 2015;43(8):1529–1541. 10.1007/s10802-015-0041-8 26081013

[4] Marini VA Stickle TR: Evidence for deficits in reward responsivity in antisocial youth with callous-unemotional traits. 2010;1(4):218–229. 10.1037/a0017675 22448665

[5] Ray JV Thornton LC Frick PJ: Impulse Control and Callous-Unemotional Traits Distinguish Patterns of Delinquency and Substance Use in Justice Involved Adolescents: Examining the Moderating Role of Neighborhood Context. 2016;44(3):599–611. 10.1007/s10802-015-0057-0 26201308

[6] Wymbs BT McCarty CA King KM: Callous-unemotional traits as unique prospective risk factors for substance use in early adolescent boys and girls. 2012;40(7):1099–110. 10.1007/s10802-012-9628-5 22453863 PMC3534821

[7] Thornton LC Frick PJ Ray JV: Risky Sex, Drugs, Sensation Seeking, and Callous Unemotional Traits in Justice-Involved Male Adolescents. 2017;1–12. 10.1080/15374416.2017.1399398 29236522

[8] Blair RJ: The amygdala and ventromedial prefrontal cortex: functional contributions and dysfunction in psychopathy. 2008;363(1503):2557–2565. 10.1098/rstb.2008.0027 18434283 PMC2606709

[9] Blair RJ Colledge E Murray L: A selective impairment in the processing of sad and fearful expressions in children with psychopathic tendencies. 2001;29(6):491–498. 10.1023/A:1012225108281 11761283

[10] Mitchell DG Colledge E Leonard A: Risky decisions and response reversal: Is there evidence of orbitofrontal cortex dysfunction in psychopathic individuals? 2002;40(12):2013–2022. 10.1016/S0028-3932(02)00056-8 12207998

[11] Pardini DA Byrd AL: Perceptions of aggressive conflicts and others’ distress in children with callous-unemotional traits: ‘I’ll show you who’s boss, even if you suffer and I get in trouble’. 2012;53(3):283–291. 10.1111/j.1469-7610.2011.02487.x 22066467 PMC3822527

[12] Roose A Bijttebier P Claes L: Psychopathic traits in adolescence: Associations with the revised Reinforcement Sensitivity Theory systems. 2011;50(2):201–205. 10.1016/j.paid.2010.09.028

[13] Centifanti LC Modecki K: Throwing caution to the wind: callous-unemotional traits and risk taking in adolescents. 2013;42(1):106–119. 10.1080/15374416.2012.719460 23009655

[14] Hosker-Field AM Molnar DS Book AS: Psychopathy and risk taking: Examining the role of risk perception. 2016;91:123–132. 10.1016/j.paid.2015.11.059

[15] Kastner RM Sellbom M: Hypersexuality in college students: The role of psychopathy. 2012;53(5):644–649. 10.1016/j.paid.2012.05.005

[16] Khan R Brewer G Kim S: Students, sex, and psychopathy: Borderline and psychopathy personality traits are differently related to women and men’s use of sexual coercion, partner poaching, and promiscuity. 2017;107:72–77. 10.1016/j.paid.2016.11.027

[17] Centifanti LCM Modecki KL MacLellan S: Driving Under the Influence of Risky Peers: An Experimental Study of Adolescent Risk Taking. 2014 10.1111/jora.12187

[18] Essau CA Anastassiou-Hadjicharalambous X Muñoz LC: Psychometric properties of the Spence Children’s Anxiety Scale (SCAS) in Cypriot children and adolescents. 2011;42(5):557–568. 10.1007/s10578-011-0232-7 21630020

[19] Kimonis ER Frick PJ Skeem JL: Assessing callous-unemotional traits in adolescent offenders: validation of the Inventory of Callous-Unemotional Traits. 2008;31(3):241–252. 10.1016/j.ijlp.2008.04.002 18514315

[20] Roose A Bijttebier P Decoene S: Assessing the affective features of psychopathy in adolescence: a further validation of the inventory of callous and unemotional traits. 2010;17(1):44–57. 10.1177/1073191109344153 19797326

[21] Lejuez CW Read JP Kahler CW: Evaluation of a behavioral measure of risk taking: The Balloon Analogue Risk Task (BART). 2002;8(2):75–84. 10.1037/1076-898X.8.2.75 12075692

[22] Chein J Albert D O’Brien L: Peers increase adolescent risk taking by enhancing activity in the brain’s reward circuitry. 2011;14(2):F1–F10. 10.1111/j.1467-7687.2010.01035.x 21499511 PMC3075496

[23] Fanti KA Kimonis ER Hadjicharalambous MZ: Do neurocognitive deficits in decision making differentiate conduct disorder subtypes? 2016;25(9):989–996. 10.1007/s00787-016-0822-9 26832949

[24] JASP Team: JASP (Version 0.8.2).2018.

[25] Rouder JN Morey RD: Default Bayes Factors for Model Selection in Regression. 2012;47(6):877–903. 10.1080/00273171.2012.734737 26735007

[26] Kass RE Raftery AE: Bayes Factors. 1995;90(430):773–795. 10.2307/2291091

[27] Muñoz LC Qualter P Padgett G: Empathy and bullying: Exploring the influence of callous-unemotional traits. 2011;42(2):183–196. 10.1007/s10578-010-0206-1 20886285

[28] Lui JH Barry CT Sacco DF: Callous-unemotional traits and empathy deficits: Mediating effects of affective perspective-taking and facial emotion recognition. 2016;30(6):1049–1062. 10.1080/02699931.2015.1047327 26192073

[29] Centifanti LCM Negen J: Dataset 1 in: Strong evidence that callous–unemotional traits are not related to risk-taking task performance. 2018 Data Source 10.12688/f1000research.14623.1 PMC5954327 29862023

[30] Swogger MT Walsh Z Lejuez CW: Psychopathy and Risk Taking among Jailed Inmates. 2010;37(4):439–452. 10.1177/0093854810361617 20419073 PMC2856971

[31] Scott S Knapp M Henderson J: Financial cost of social exclusion: follow up study of antisocial children into adulthood. 2001;323(7306): 191. 10.1136/bmj.323.7306.191 11473907 PMC35269

[32] McMahon RJ Witkiewitz K Kotler JS: Predictive validity of callous-unemotional traits measured in early adolescence with respect to multiple antisocial outcomes. 2010;119(4):752–63. 10.1037/a0020796 20939651 PMC3760169

[33] Muñoz LC Frick PJ: The reliability, stability, and predictive utility of the self-report version of the Antisocial Process Screening Device. 2007;48(4):299–312. 10.1111/j.1467-9450.2007.00560.x 17669220

