# Supplementary material for: Strong evidence that callous–unemotional traits are not related to risk-taking task performance
Source: F1000Res. 2018 Apr 26;7:502. [Version 1] doi: 10.12688/f1000research.14623.1 (PMC5954327; doi:10.12688/f1000research.14623.1)
Supplement: Peer review history

# Peer review: Referee response for version 1

**DOI:** 10.5256/f1000research.15914.r33543

**Published:** 2018-05-11

**Authors:** Timothy R. Stickle, Andrew Gill, Ashley Dobbins, Amanda Falcon

**Affiliations:** 1 Department of Psychological Science, University of Vermont, Burlington, VT, USA; 2 University of Vermont, Burlington, VT, USA

This is an interesting research note and is likely to contribute to the literature. There are, however, several areas that need elaboration or clarification to better communicate the findings and their contribution. 

 Use of Bayesian analysis in a reasonably large sample adds strength to the authors interpretation of null findings. 

 Clarification about some detail of the Bayesian analysis for the reader would be helpful and should include:

 How was the prior selected and what was used for the prior distribution? 

 Was it a noninformative or an informative prior? 

 Was sensitivity analysis conducted to gauge the effect of the prior? 

 It is worth noting that the sample is reasonably large, so the effect of the prior on at least some parameters may be small. Nevertheless, it would be helpful for the authors to note the effect of sample size on prior and posterior probabilities. 

 In the first paragraph of the Discussion section, the authors make a strong declarative statement that the results “…rule out a specific theory about why CU traits are related to risky real-world behaviors... People with CU traits are not more likely to engage in risky behavior in a lab setting, so real-world risky behaviors are unlikely to be driven by risk-seeking for its own sake.” 

 Although the following statements regarding caution in generalizing reward and punishment processing in laboratory tasks to behavior outside the lab clearly are warranted, the above statements seem a step too far. That is, the results clearly cast some doubt on the links and mechanisms of risk. The notion that the finding is conclusive, however, does not seem warranted. Rather, it is recommended that the authors use language to indicate that the current results fail to support links between the specific measure of risk used here and CU traits. It is a reasonable inference to note that the presence of CU traits alone and documented mechanisms of reward and punishment processing are not de facto predictors of risk. It seems more defensible to state something more akin to noting that the currents results call for reexamination of the mechanisms thought to link CU traits and risky behaviors.  

 In extending these recommended comments, it would also be helpful for the authors to synthesize the discussion paragraphs a bit more in their discussion of the multifaceted/multidimensional nature of psychopathy and the implications of the broader psychopathy construct in understanding risk and its mechanisms. 

 Overall, the paper is interesting and the above comments are offered in the spirit of strengthening how the results are communicated.

We have read this submission. We believe that we have an appropriate level of expertise to confirm that it is of an acceptable scientific standard.

# Peer review: Referee response for version 1

**DOI:** 10.5256/f1000research.15914.r33540

**Published:** 2018-05-08

**Authors:** Carlo Garofalo

**Affiliations:** 1 Department of Developmental Psychology, Tilburg University, Tilburg, Netherlands

Thanks for the opportunity to review this manuscript. The study described has important strengths, such as the large sample, the use of well-validated assessment methods, and the sophisticated statistical techniques. I also extremely appreciate the fact that full data are made available, something that happens too rarely to the detriment of the field. The topic is certainly an important one, and one that historically attracts speculations that permeates popular beliefs on the nature and dynamics of callous-unemotional traits. I therefore think that the publication of (null) findings like this is imperative as to not fuel misconceptions on the topic.

 While I am therefore clearly positive regarding this submission, I also list below some issues that occurred to me while reading the manuscript, which I think may deserve further elaboration to strengthen the contribution of this study to the literature. I suspect that the writing was constrained by length limits, which could make it impossible to address my suggestions thoroughly, but I thought I would nevertheless mention what I believe could further improve this well-executed and well-written study.

1. It is clear to me that the findings do not support the hypothesis that CU traits are related to risk taking. But I could not tell: who proposed this link, and whether this hypothesis also guided the present research. I think it would help situate the present study within the broader literature on CU if the manuscript could (briefly) refer whether the CU-risk taking link was just one that ‘makes sense’, or one that is central in existing theories of CU (e.g., in the opening of the discussion, it is stated that the present study rules out a ‘specific theory’, but it is unclear which theory is referred to). I think this is important because such theories would have to be re-considered in light of this null finding (as opposed to the scenario in which the CU-risk taking link is more of a popular misconceptions with no footing in scholarly work). Relatedly, as I find the alternative explanation (i.e., ‘a different method of valuing certain outcomes’) equally – if not more – compelling, I think it would be helpful to know whether this possibility was already acknowledged before the study was conducted, or followed the null findings that did not support the initial hypothesis.
2. A second conceptual clarification that may be needed concern the overlap and dissociation between reward/punishment sensitivity and risk-taking. This seems relevant to understand specific abnormalities related to CU traits and what they can mean. Related to this – it looks like CU traits were also not related in punishment sensitivity, if I am not misunderstanding. If that is the case, how does this null finding aligns with existing theories of psychopathy such as the low-fear hypothesis or the response modulation model?
3. For non-familiar readers (as myself), it may be worth expanding on how the Bayesian framework adopted allows for an interpretation of the null finding in a way that frequentist methods would not (also just a brief mention of why the overall fit can be considered strong evidence of null hypothesis).
4. I cannot help but wonder what the results look like if the ICU is examined at a sub-scale level. I do not expect differences, but I think it is important to rule out that the overall null effect is not due to differential associations between ICU subscales and risk-taking.

I have read this submission. I believe that I have an appropriate level of expertise to confirm that it is of an acceptable scientific standard.
